# Supplementary material for: Viral potential to modulate microbial methane metabolism varies by habitat
Source: Nat Commun. 2024 Feb 29;15:1857. doi: 10.1038/s41467-024-46109-x (PMC10904782; doi:10.1038/s41467-024-46109-x)
Supplement: Supplementary file 1 — Supplementary Information [file 41467_2024_46109_MOESM1_ESM.pdf]

Supplementary Discussion:

Viruses encode seven AMGs exclusively participating in methane metabolism pathway

Through analyzing 982 published metagenomes, we discovered 24 distinct AMGs being able to putatively participate in methane metabolism pathway (MMP), including 17 genes that could also be involved in other types of metabolic pathways and seven genes (i.e., *mtrA*, *pmoC*, *fwdF*, *fae*, *cofE*, *cofF*, and *frhB*) that exclusively participate in MMP (Supplementary Fig. S1 & Data 5). Of the later seven genes, *mtrA* genes were identified on three viral contigs recovered from three different rumen samples[1], a typical host-associated methanogenic habitat (Supplementary Data 5). The three viral contigs belonged to the same viral population (97.4–97.8% genomic identities among each other) and shared an identical *mtrA* gene sequence (Fig. 1B). The finding of an identical gene among different strains within a viral population could be due to horizontal gene transfer within the viral population and/or due to no mutation occurring on the gene after a population purification. We also recovered 25 virus-encoded *pmoC* genes (*vPmoC*; from freshwater lake water), including 23 lake viral genes that were identical to the previously-reported *vPmoC* genes from the same freshwater lake[2] (Supplementary Fig. S5B). These *pmoC* genes were from 25 viral contigs belonging to seven viral populations (Supplementary Data 5). The *fwdF* gene was identified from a rumen viral contig with a complete genome, while the *fae* gene was recovered from a marine sediment viral contig (Supplementary Data 5). The *cofE* gene was identified from 17 rumen viral contigs (belonging to seven viral populations), while the *cofF* genes were found in seven viral contigs (belonging to five viral populations) from four different habitats (rumen, lake water, marine water, and marine sediments) and the *frhB* gene was recovered on five viral contigs (belonging to three viral populations) from rumen and marine sediment (Supplementary Data 5).

Ten AMGs can potentially modulate methane production

In addition to the six exclusive MM AMGs (i.e., *mtrA*, *fwdF*, *fae*, *cofE*, *cofF*, and *frhB*), four of the 17 non-exclusive MM AMGs (i.e., *ackA*, *pta*, *cooS*, and *glyA*, that are able to participate in metabolic pathways other than MMP) were also involved in the pathway of methanogenesis from CO_2 or acetate (Fig. 1A; Supplementary Fig. S1 & Data 5). The *ackA* and *pta* genes encode acetate kinase and phosphate acetyltransferase, respectively and can potentially mediate the first two steps of methanogenesis from acetate, by catalyzing the metabolic reactions from acetate to acetyl phosphate, and then to acetyl coenzyme A[3] (Supplementary

Fig. S1 & Data 5). The carbon monoxide dehydrogenase gene (*cooS*) catalyzes the oxidation of CO to CO_2[4], which can be used as substrate for methanogenesis from CO_2[5], or in itself, could be a step of methanogenesis pathway using CO as the substrate[6]. While the *glyA* gene encodes serine hydroxymethyltransferase that can catalyze the reaction for producing methylene-THMPT, an intermediate in methanogenesis from CO_2, from serine and tetrahydromethanopterin (Supplementary Fig. S1 & Data 5).

Distinct viral communities detected in bulk metagenomes versus viromes

Comparison between the viromes and bulk metagenomes found that 90% (79 of 88) of the virome-recovered vOTUs were also identified in the bulk metagenomes, and that the latter recovered 35.6 times more (3,137 versus 88) vOTUs (Supplementary Fig. S9B & Data 11). Ordination analysis revealed that viromes and bulk metagenomes recovered significantly ($p = 0.003$) different viral communities (Fig. 3D). These results indicate that most of the recoverable VLS viruses come from intracellular spaces that were captured by the bulk metagenomes, in agreement with previous reports that temperate viruses are diverse and abundant in various environments[7-9]. However, we cannot eliminate the possibility that our viromes might have only captured a subset of VLS extracellular viruses (e.g., some extracellular viruses might have been absorbed to the sediment particles which were removed from viromes via filtering, but captured in bulk metagenomes). Obtaining less vOTUs from viromes than bulk metagenomes is not unusual for particle-associated samples, and is consistent with previous studies from soils (e.g., only 53 vOTUs were recovered from 7 soil viromes[10]), while the soil bulk metagenomes, in a same project, obtained an average of 3.5 times more vOTUs per sample than viromes, which though would have benefited from deeper sequencing efforts for the viral DNA[11]).

VLS virus encodes *bfr* gene to impact iron metabolism

Iron is an essential element and is the most common redox active metal in proteins (e.g., heme and iron-sulfur prosthetic groups)[12,13]. We identified a VLS vOTU which encoded the AMG *bfr* (Fig. 4C), a bacterioferritin that oxidizes Fe^{2+} to Fe^{3+}[14]. Iron is essential for numerous metabolic processes[12], including MM[15-17]. Phylogenetic analysis revealed that this virus-encoded *bfr* gene (*vBfr*) might have been transferred from an archaeon within the phylum Thermoproteota (Fig. 4D; Supplementary Fig. S11), which was the putative host of this virus (Supplementary Data 12 & Data 15) and the most dominant phylum in VLS (Fig. 4B; Supplementary Fig. S10). Notably, genomic annotations found that the predicted host of this

virus contained 62 genes (39 distinct genes after dereplication) involved in the MMP (Supplementary Data 13) and thus was probably a methanogen. Further exploration of the conserved amino-acid domain and evolutionary dynamics within species and across lineages found that the virus-encoded Bfr was likely functional and under purification selection (*pN/pS* = 0; average *dN/dS* = 0.114) (Supplementary Fig. S12A, Data 15, & Data 16). Overall, we propose that the VLS virus, which infected a methanogenic member of the phylum Thermoproteota, might be able to functionally modulate its hosts' iron metabolism, and thus had the potential to indirectly impact MM.

Supplementary Figures:

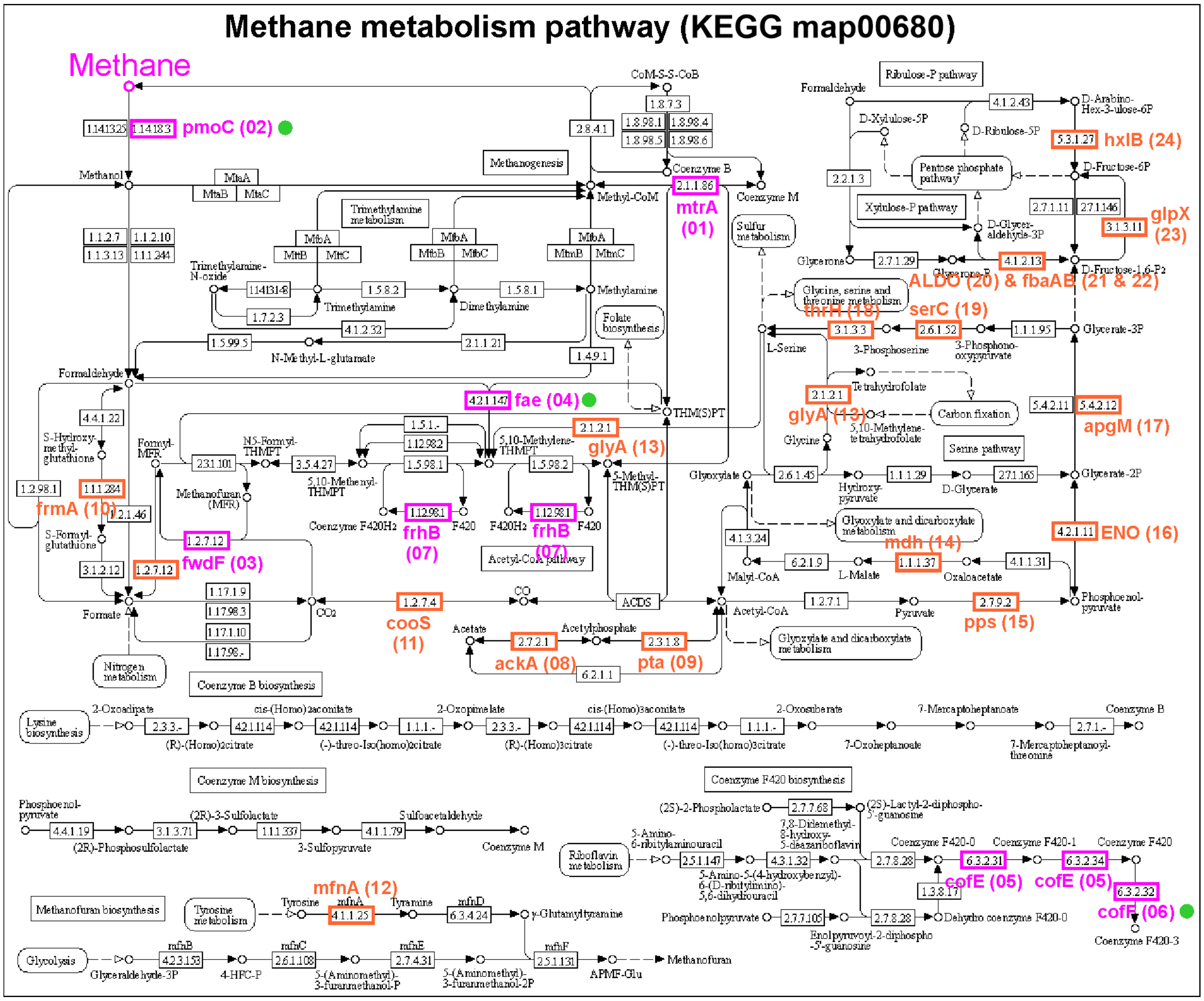

Figure S1. Viral participations in MMP. The enzymes are highlighted by rectangles (either orange or purple) if one of their subunits could be potentially encoded by at least one AMG, which is indicated next to the rectangles. A total of 24 methane metabolism AMGs were discovered. The IDs (numbers) in the parentheses after each AMG name indicate the AMG's order, by which the genome maps of a representative viral contig carrying each of the AMGs are illustrated in Supplementary Fig. S2 and they were summarized in Supplementary Data 5. The seven AMGs *mtrA*, *pmoC*, *fwdF*, *fae*, *cofE*, *cofF*, and *frhB*, that exclusively participate in MMP, are highlighted in purple; while the other 17 AMGs that can participate other types of metabolism pathways are colored in orange. Only three of the 24 AMGs (i.e., *pmoC*, *cofF*, and *fae* genes) have been reported previously and are indicated by a green circle next to the AMG name. The map of MMP is adapted from KEGG's map00680 (https://www.genome.jp/pathway/map00680). MMP, methane metabolism pathway.

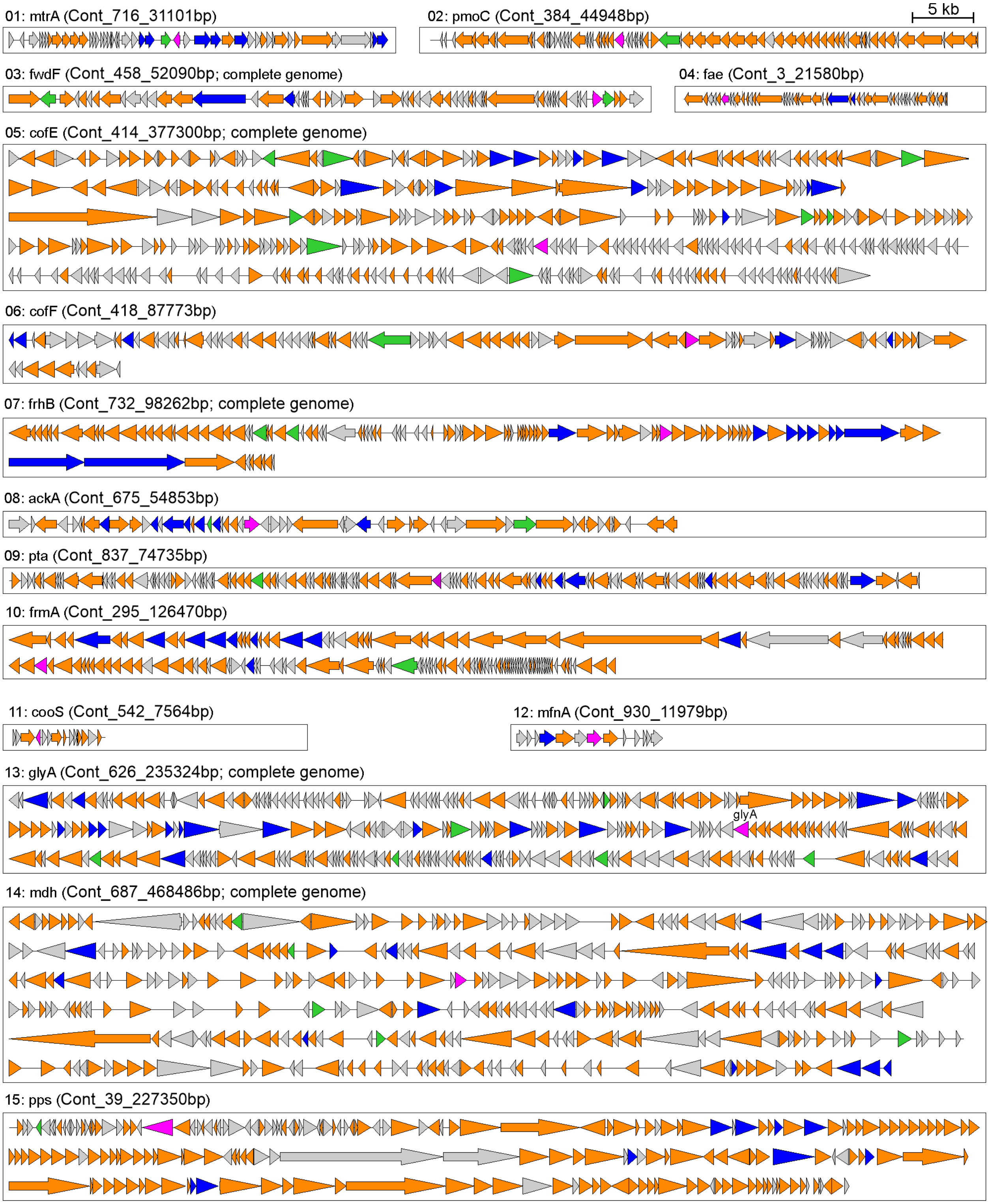
01: mtrA (Cont_716_31101bp)
02: pmoC (Cont_384_44948bp)
5 kb
03: fwdF (Cont_458_52090bp; complete genome)
04: fae (Cont_3_21580bp)
05: cofE (Cont_414_377300bp; complete genome)
06: cofF (Cont_418_87773bp)
07: frhB (Cont_732_98262bp; complete genome)
08: ackA (Cont_675_54853bp)
09: pta (Cont_837_74735bp)
10: frmA (Cont_295_126470bp)
11: cooS (Cont_542_7564bp)
12: mfnA (Cont_930_11979bp)
13: glyA (Cont_626_235324bp; complete genome)
glyA
14: mdh (Cont_687_468486bp; complete genome)
15: pps (Cont_39_227350bp)

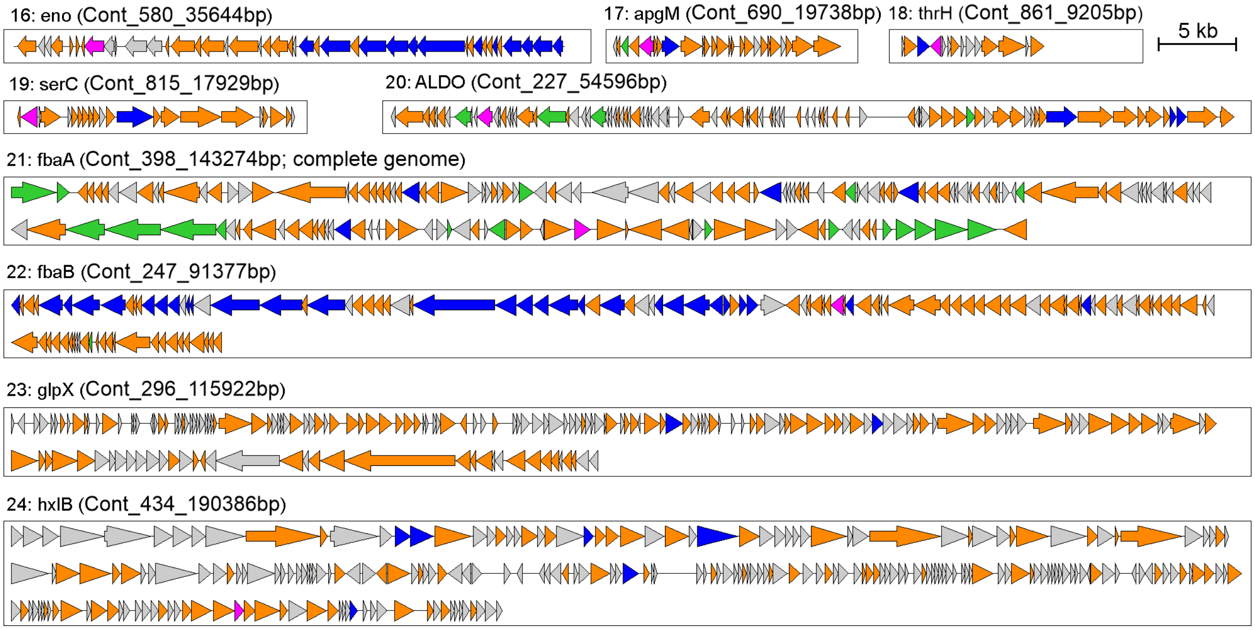

Figure S2. Genome maps of representative viral contigs containing the 24 MM AMGs. These 24 MM AMGs participated in 25 reactions in the MMP, as indicated by the IDs (numbers) and AMG names that are described both on the top of each genome map in this figure and next to each of the enzymatic reactions in Supplementary Fig. S1. For each of the 24 AMGs, one viral contig was selected as an example to illustrate the genome map and AMG position (see Methods). Complete genomes (n = 6) are indicated after the contig name. CheckV was used to assess host-virus boundaries and remove potential host fractions on the viral contig. Genes were marked by five colors to illustrate AMGs (purple), phage genes (orange), phage hallmark genes (blue), potential cellular genes (green), and hypothetical protein genes (grey). AMGs were detected by both the tool VIBRIANT and verified by subsequent manual inspections; the latter three groups of genes were classified by comparing their predicted protein sequences to those in VIBRIANT's, CheckV's, and VirSorter's databases. Genes were also annotated by comparing them to KEGG and PFAM databases. Genes were marked as "phage genes" if they were matched to the genes of viruses in the tested databases, while were considered "hypothetical" if they had no hit to a sequence in any tested databases. MM, methane metabolism; MMP, methane metabolism pathway.

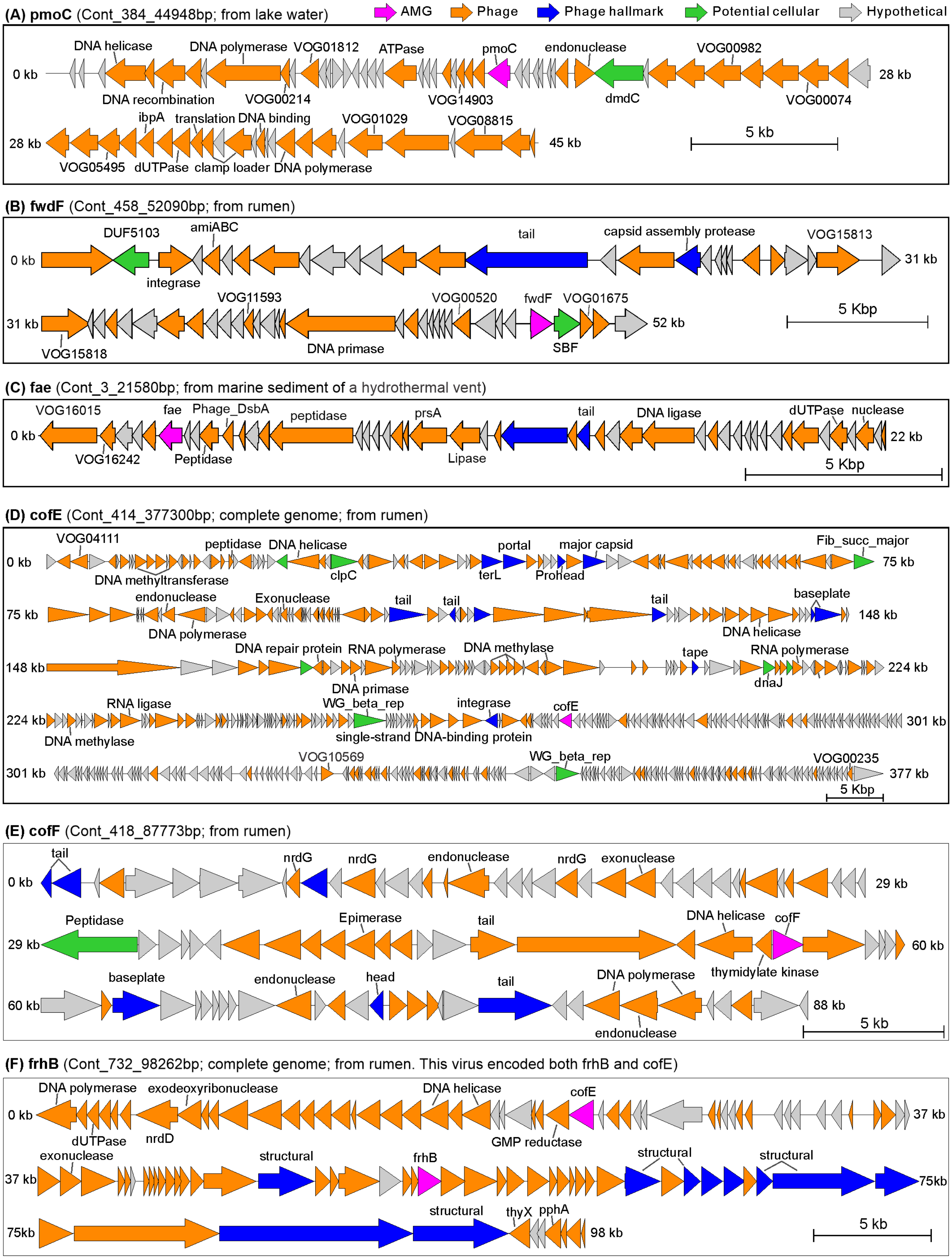
(A) pmoC (Cont_384_44948bp; from lake water)
AMG
Phage
Phage hallmark
Potential cellular
Hypothetical
DNA helicase
DNA polymerase
VOG01812
ATPase
pmoC
endonuclease
VOG00982
0 kb
28 kb
DNA recombination
VOG00214
VOG14903
dmdC
VOG00074
ibpA
translation
DNA binding
VOG01029
VOG08815
28 kb
45 kb
5 kb
VOG05495
dUTPase
clamp loader
DNA polymerase
(B) fwdF (Cont_458_52090bp; from rumen)
DUF5103
amiABC
tail
capsid assembly protease
VOG15813
0 kb
31 kb
integrase
VOG11593
VOG00520
fwdF
VOG01675
31 kb
52 kb
5 Kbp
VOG15818
DNA primase
SBF
(C) fae (Cont_3_21580bp; from marine sediment of a hydrothermal vent)
VOG16015
fae
Phage_DsbA
peptidase
prsA
tail
DNA ligase
dUTPase
nuclease
0 kb
22 kb
VOG16242
Peptidase
Lipase
5 Kbp
(D) cofE (Cont_414_377300bp; complete genome; from rumen)
VOG04111
peptidase
DNA helicase
portal
major capsid
Fib_succ_major
0 kb
75 kb
DNA methyltransferase
clpC
terL
Prohead
endonuclease
Exonuclease
tail
tail
tail
baseplate
75 kb
148 kb
DNA polymerase
DNA helicase
DNA repair protein
RNA polymerase
DNA methylase
tape
RNA polymerase
148 kb
224 kb
DNA primase
dnaJ
RNA ligase
WG_beta_rep
integrase
cofE
224 kb
301 kb
DNA methylase
single-strand DNA-binding protein
VOG10569
WG_beta_rep
VOG00235
301 kb
377 kb
5 Kbp
(E) cofF (Cont_418_87773bp; from rumen)
tail
nrdG
nrdG
endonuclease
nrdG
exonuclease
0 kb
29 kb
Peptidase
Epimerase
tail
DNA helicase
cofF
29 kb
60 kb
thymidylate kinase
baseplate
endonuclease
head
tail
DNA polymerase
60 kb
88 kb
endonuclease
5 kb
(F) frhB (Cont_732_98262bp; complete genome; from rumen. This virus encoded both frhB and cofE)
DNA polymerase
exodeoxyribonuclease
DNA helicase
cofE
0 kb
37 kb
dUTPase
nrdD
GMP reductase
exonuclease
structural
frhB
structural
structural
37 kb
75kb
structural
thyX
pphA
75kb
98 kb
5 kb

Figure S3. Genome maps of the representative viral contigs containing the six AMGs that exclusively participate in MMP. The six AMGs comprised: (A) *pmoC*, (B) *fwdF*, (C) *fae*, (D) *cofE*, (E) *cofF*, and (F) *frhB* genes. The genome map of another exclusive MM AMG *mtrA* is illustrated in Fig. 1B. The genome composition of these viral contigs is also provided in Supplementary Fig. S2, which, however, did not include the gene annotation information. Genes were marked by five colors to illustrate AMGs (purple), phage genes (orange), phage hallmark genes (blue), potential cellular genes (green), and hypothetical protein genes (grey), as described in Supplementary Fig. S2. Kb, kilobases.

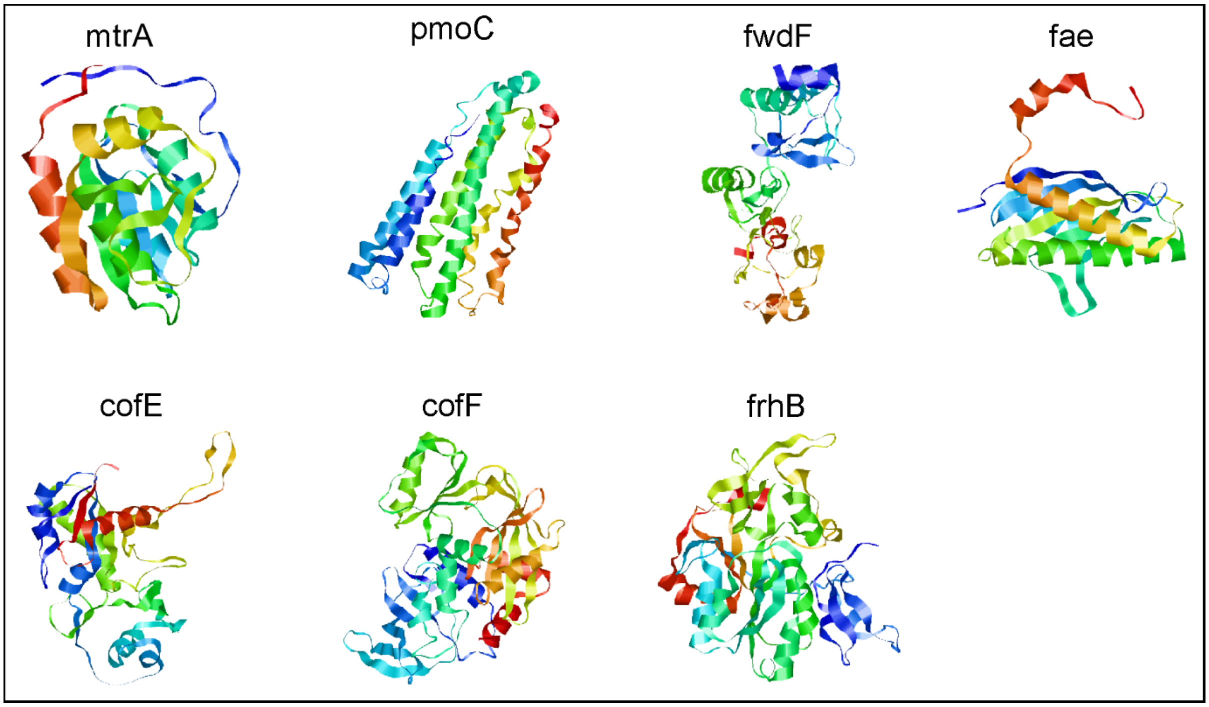

Figure S4. Predicted three-dimensional (3D) structures of seven AMG products. The seven AMGs comprised: *mtrA*, *pmoC*, *fwdF*, *fae*, *cofE*, *cofF*, and *frhB* genes. All these AMG products are linked to their closest template protein with 100% confidence score (except *fwdF* with 99%), by phyre2. Additional information of the protein structure modelling for all 24 MM AMGs is summarized in Supplementary Data 5.

(A) *mtrA* gene

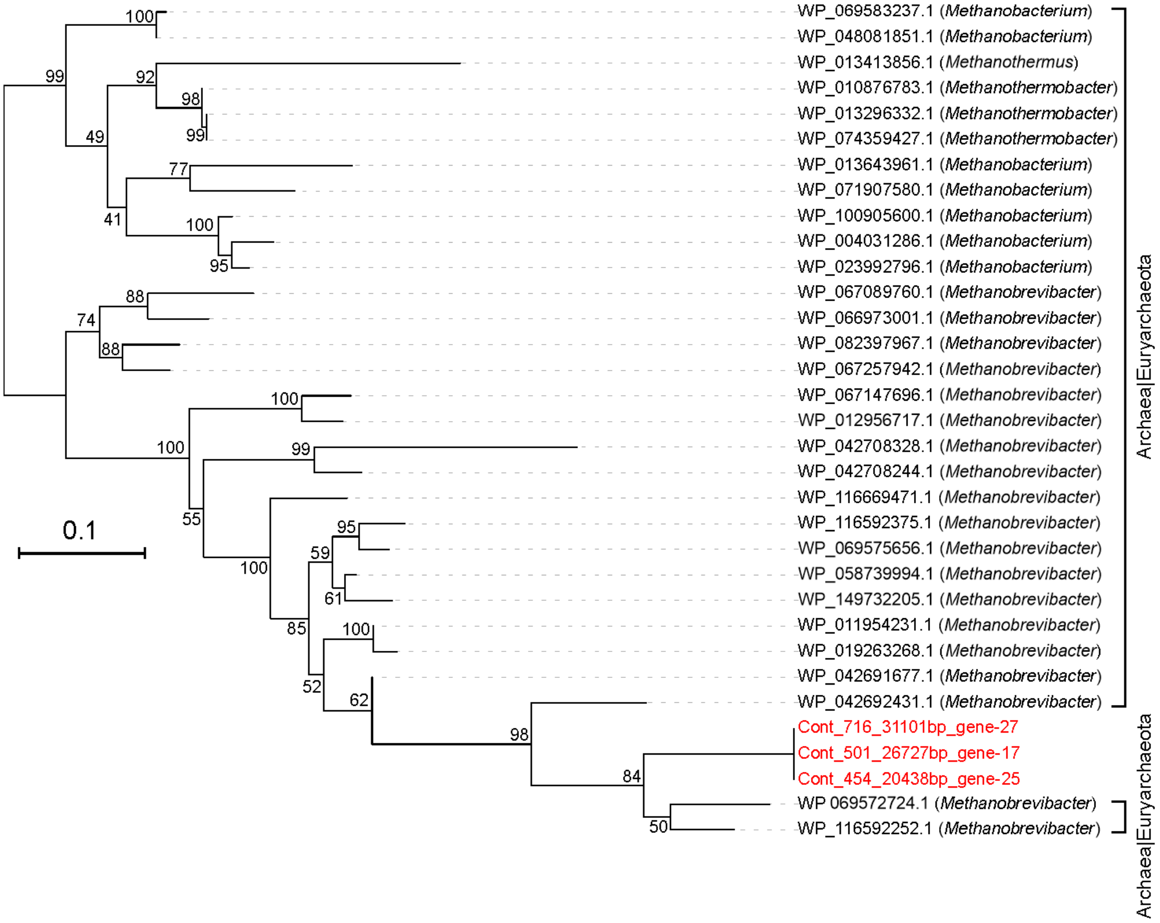

WP_069583237.1 (Methanobacterium)
WP_048081851.1 (Methanobacterium)
WP_013413856.1 (Methanothermus)
WP_010876783.1 (Methanothermobacter)
WP_013296332.1 (Methanothermobacter)
WP_074359427.1 (Methanothermobacter)
WP_013643961.1 (Methanobacterium)
WP_071907580.1 (Methanobacterium)
WP_100905600.1 (Methanobacterium)
WP_004031286.1 (Methanobacterium)
WP_023992796.1 (Methanobacterium)
WP_067089760.1 (Methanobrevibacter)
WP_066973001.1 (Methanobrevibacter)
WP_082397967.1 (Methanobrevibacter)
WP_067257942.1 (Methanobrevibacter)
WP_067147696.1 (Methanobrevibacter)
WP_012956717.1 (Methanobrevibacter)
WP_042708328.1 (Methanobrevibacter)
WP_042708244.1 (Methanobrevibacter)
WP_116669471.1 (Methanobrevibacter)
WP_116592375.1 (Methanobrevibacter)
WP_069575656.1 (Methanobrevibacter)
WP_058739994.1 (Methanobrevibacter)
WP_149732205.1 (Methanobrevibacter)
WP_011954231.1 (Methanobrevibacter)
WP_019263268.1 (Methanobrevibacter)
WP_042691677.1 (Methanobrevibacter)
WP_042692431.1 (Methanobrevibacter)
Cont_716_31101bp_gene-27
Cont_501_26727bp_gene-17
Cont_454_20438bp_gene-25
WP 069572724.1 (Methanobrevibacter)
WP_116592252.1 (Methanobrevibacter)
Archaea|Euryarchaeota
Archaea|Euryarchaeota
0.1

(B) *pmoC* gene

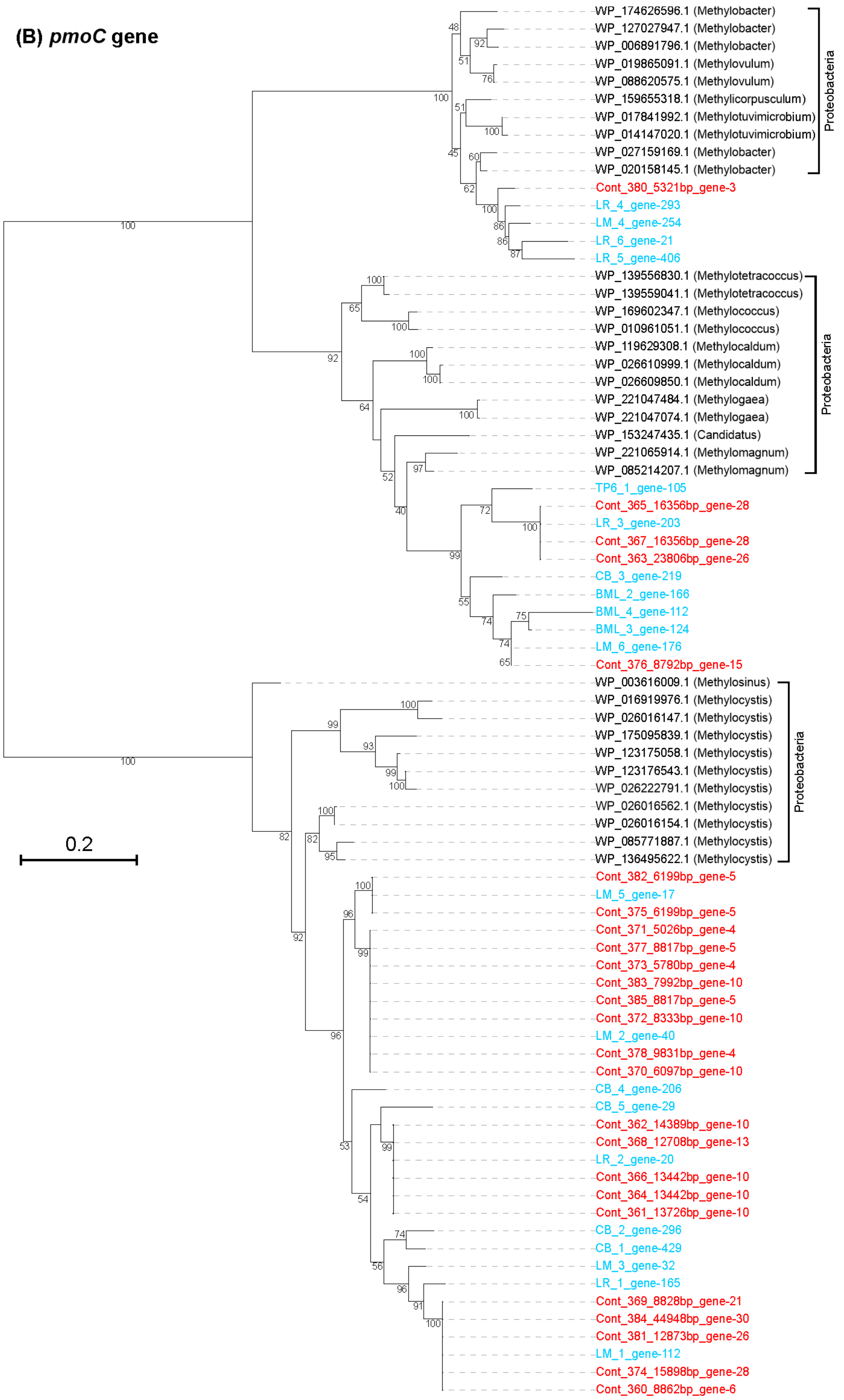
WP_174626596.1 (Methylobacter)
WP_127027947.1 (Methylobacter)
WP_006891796.1 (Methylobacter)
WP_019865091.1 (Methylovulum)
WP_088620575.1 (Methylovulum)
WP_159655318.1 (Methylicorpusculum)
WP_017841992.1 (Methylotuvimicrobium)
WP_014147020.1 (Methylotuvimicrobium)
WP_027159169.1 (Methylobacter)
WP_020158145.1 (Methylobacter)
Proteobacteria
Cont_380_5321bp_gene-3
LR_4_gene-293
LM_4_gene-254
LR_6_gene-21
LR_5_gene-406
WP_139556830.1 (Methylotetracoccus)
WP_139559041.1 (Methylotetracoccus)
WP_169602347.1 (Methylococcus)
WP_010961051.1 (Methylococcus)
WP_119629308.1 (Methylocaldum)
WP_026610999.1 (Methylocaldum)
WP_026609850.1 (Methylocaldum)
WP_221047484.1 (Methylogaea)
WP_221047074.1 (Methylogaea)
WP_153247435.1 (Candidatus)
WP_221065914.1 (Methylomagnum)
WP_085214207.1 (Methylomagnum)
Proteobacteria
TP6_1_gene-105
Cont_365_16356bp_gene-28
LR_3_gene-203
Cont_367_16356bp_gene-28
Cont_363_23806bp_gene-26
CB_3_gene-219
BML_2_gene-166
BML_4_gene-112
BML_3_gene-124
LM_6_gene-176
Cont_376_8792bp_gene-15
WP_003616009.1 (Methylosinus)
WP_016919976.1 (Methylocystis)
WP_026016147.1 (Methylocystis)
WP_175095839.1 (Methylocystis)
WP_123175058.1 (Methylocystis)
WP_123176543.1 (Methylocystis)
WP_026222791.1 (Methylocystis)
WP_026016562.1 (Methylocystis)
WP_026016154.1 (Methylocystis)
WP_085771887.1 (Methylocystis)
WP_136495622.1 (Methylocystis)
Proteobacteria
Cont_382_6199bp_gene-5
LM_5_gene-17
Cont_375_6199bp_gene-5
Cont_371_5026bp_gene-4
Cont_377_8817bp_gene-5
Cont_373_5780bp_gene-4
Cont_383_7992bp_gene-10
Cont_385_8817bp_gene-5
Cont_372_8333bp_gene-10
LM_2_gene-40
Cont_378_9831bp_gene-4
Cont_370_6097bp_gene-10
CB_4_gene-206
CB_5_gene-29
Cont_362_14389bp_gene-10
Cont_368_12708bp_gene-13
LR_2_gene-20
Cont_366_13442bp_gene-10
Cont_364_13442bp_gene-10
Cont_361_13726bp_gene-10
CB_2_gene-296
CB_1_gene-429
LM_3_gene-32
LR_1_gene-165
Cont_369_8828bp_gene-21
Cont_384_44948bp_gene-30
Cont_381_12873bp_gene-26
LM_1_gene-112
Cont_374_15898bp_gene-28
Cont_360_8862bp_gene-6
0.2

(C) *fwdF* gene

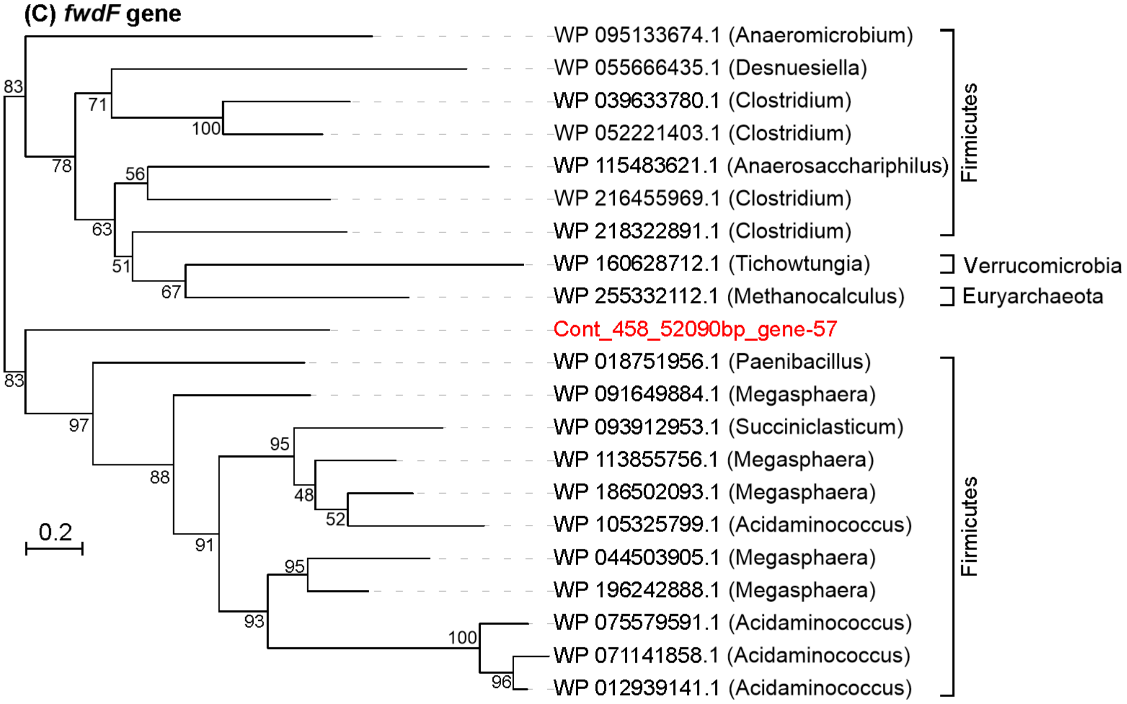

(D) *fae* gene

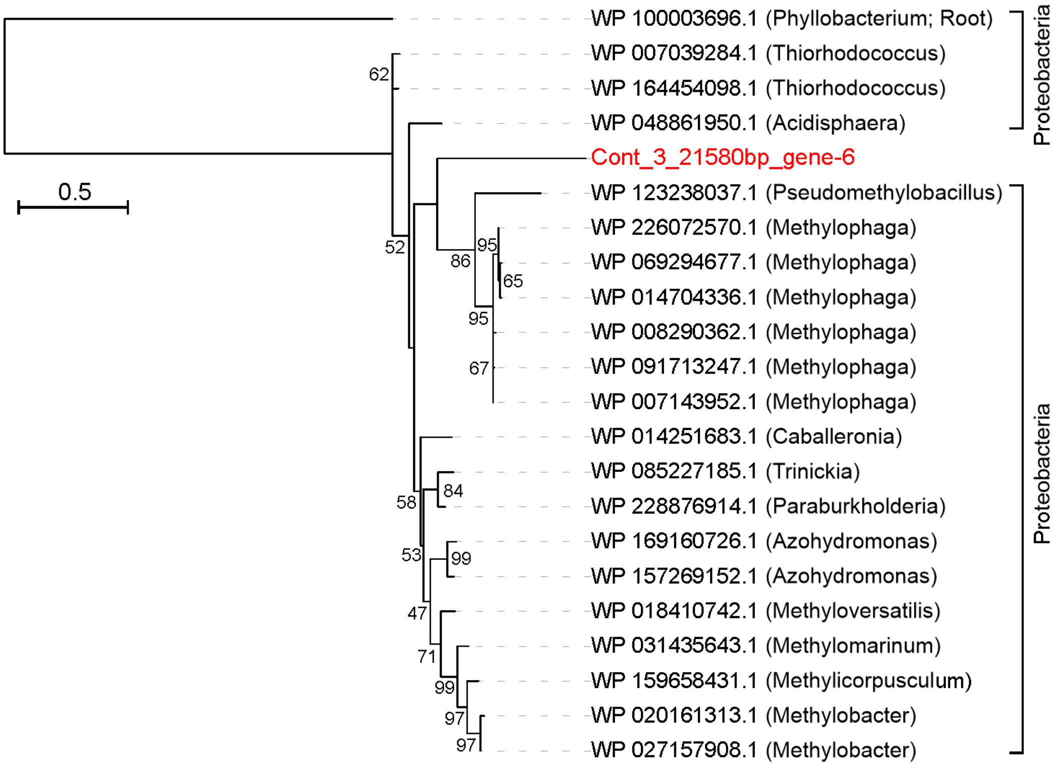

(E) *cofE* gene

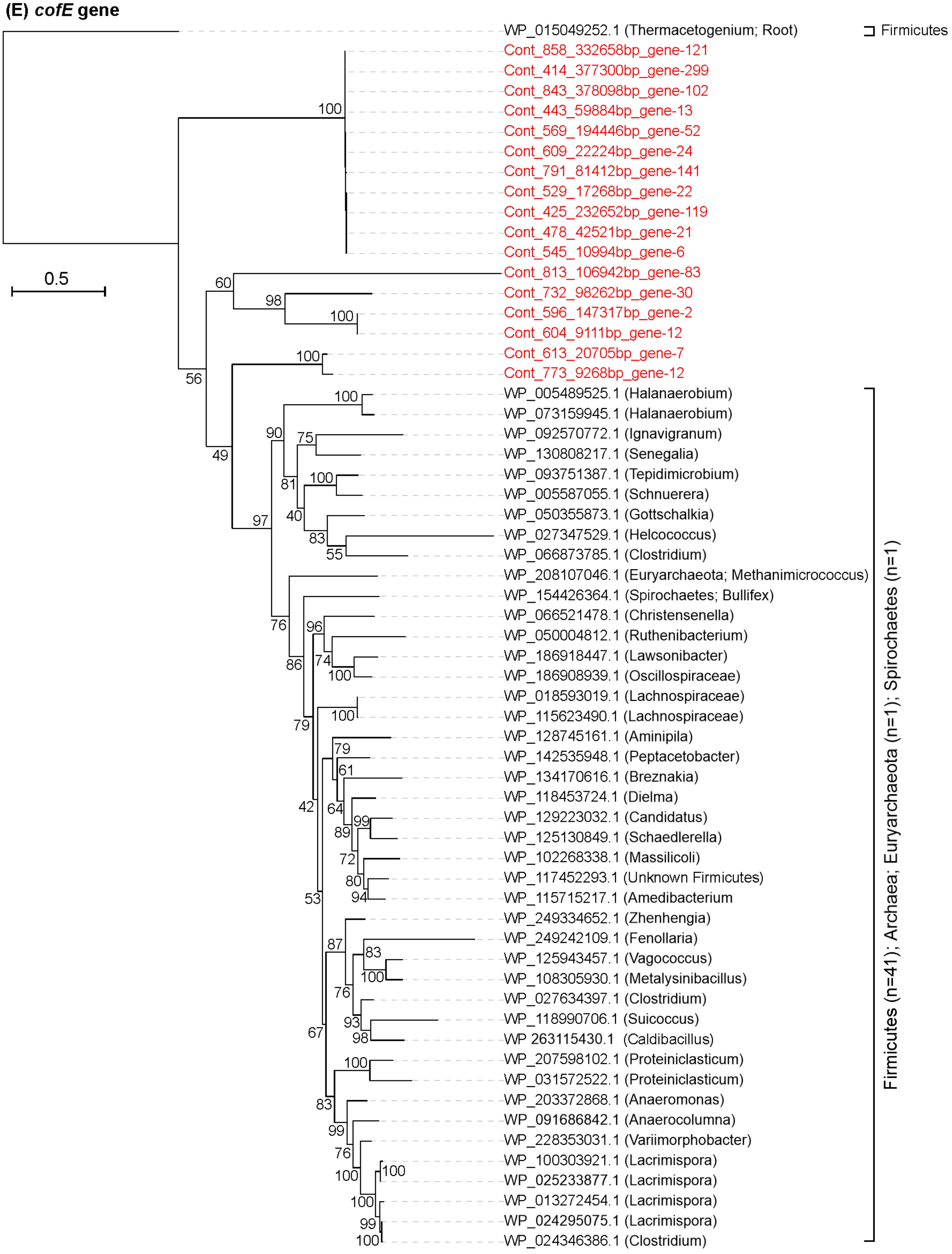

0.5
WP_015049252.1 (Thermacetogenium; Root)
Firmicutes
Cont_858_332658bp_gene-121
Cont_414_377300bp_gene-299
Cont_843_378098bp_gene-102
Cont_443_59884bp_gene-13
Cont_569_194446bp_gene-52
Cont_609_22224bp_gene-24
Cont_791_81412bp_gene-141
Cont_529_17268bp_gene-22
Cont_425_232652bp_gene-119
Cont_478_42521bp_gene-21
Cont_545_10994bp_gene-6
Cont_813_106942bp_gene-83
Cont_732_98262bp_gene-30
Cont_596_147317bp_gene-2
Cont_604_9111bp_gene-12
Cont_613_20705bp_gene-7
Cont_773_9268bp_gene-12
WP_005489525.1 (Halanaerobium)
WP_073159945.1 (Halanaerobium)
WP_092570772.1 (Ignavigranum)
WP_130808217.1 (Senegalia)
WP_093751387.1 (Tepidimicrobium)
WP_005587055.1 (Schnuerera)
WP_050355873.1 (Gottschalkia)
WP_027347529.1 (Helcococcus)
WP_066873785.1 (Clostridium)
WP_208107046.1 (Euryarchaeota; Methanimicrococcus)
WP_154426364.1 (Spirochaetes; Bullifex)
WP_066521478.1 (Christensenella)
WP_050004812.1 (Ruthenibacterium)
WP_186918447.1 (Lawsonibacter)
WP_186908939.1 (Oscillospiraceae)
WP_018593019.1 (Lachnospiraceae)
WP_115623490.1 (Lachnospiraceae)
WP_128745161.1 (Aminipila)
WP_142535948.1 (Peptacetobacter)
WP_134170616.1 (Breznakia)
WP_118453724.1 (Dielma)
WP_129223032.1 (Candidatus)
WP_125130849.1 (Schaedlerella)
WP_102268338.1 (Massilicoli)
WP_117452293.1 (Unknown Firmicutes)
WP_115715217.1 (Amedibacterium
WP_249334652.1 (Zhenhengia)
WP_249242109.1 (Fenollaria)
WP_125943457.1 (Vagococcus)
WP_108305930.1 (Metalysinibacillus)
WP_027634397.1 (Clostridium)
WP_118990706.1 (Suicoccus)
WP 263115430.1 (Caldibacillus)
WP_207598102.1 (Proteiniclasticum)
WP_031572522.1 (Proteiniclasticum)
WP_203372868.1 (Anaeromonas)
WP_091686842.1 (Anaerocolumna)
WP_228353031.1 (Variimorphobacter)
WP_100303921.1 (Lacrimispora)
WP_025233877.1 (Lacrimispora)
WP_013272454.1 (Lacrimispora)
WP_024295075.1 (Lacrimispora)
WP_024346386.1 (Clostridium)
Firmicutes (n=41); Archaea; Euryarchaeota (n=1); Spirochaetes (n=1)

(F) *cofF* gene

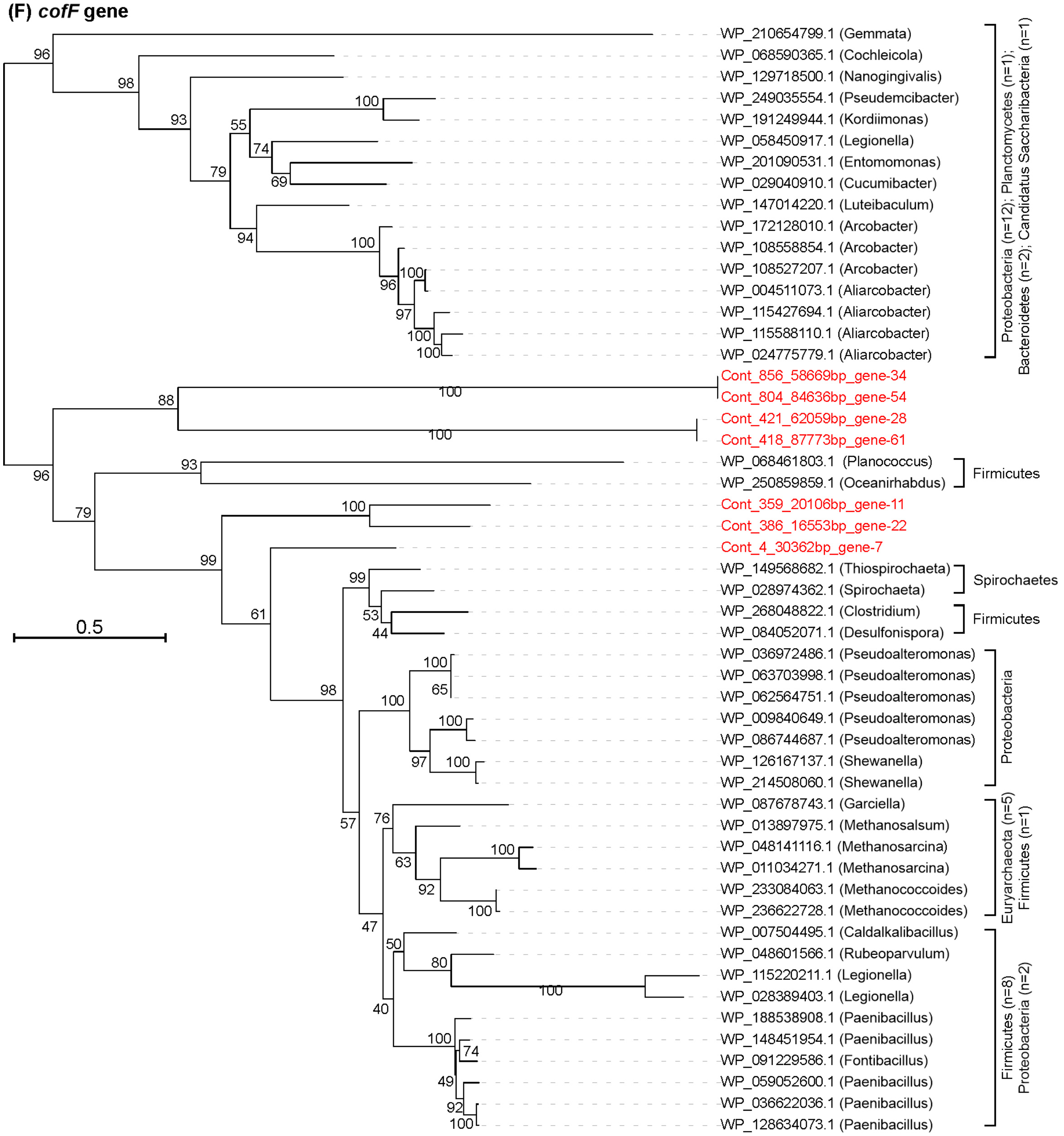

WP_210654799.1 (Gemmata)
WP_068590365.1 (Cochleicola)
WP_129718500.1 (Nanogingivalis)
WP_249035554.1 (Pseudemcibacter)
WP_191249944.1 (Kordiimonas)
WP_058450917.1 (Legionella)
WP_201090531.1 (Entomomonas)
WP_029040910.1 (Cucumibacter)
WP_147014220.1 (Luteibaculum)
WP_172128010.1 (Arcobacter)
WP_108558854.1 (Arcobacter)
WP_108527207.1 (Arcobacter)
WP_004511073.1 (Aliarcobacter)
WP_115427694.1 (Aliarcobacter)
WP_115588110.1 (Aliarcobacter)
WP_024775779.1 (Aliarcobacter)
Proteobacteria (n=12); Planctomycetes (n=1); Bacteroidetes (n=2); Candidatus Saccharibacteria (n=1)
Cont_856_58669bp_gene-34
Cont_804_84636bp_gene-54
Cont_421_62059bp_gene-28
Cont_418_87773bp_gene-61
WP_068461803.1 (Planococcus)
WP_250859859.1 (Oceanirhabdus)
Firmicutes
Cont_359_20106bp_gene-11
Cont_386_16553bp_gene-22
Cont_4_30362bp_gene-7
WP_149568682.1 (Thiospirochaeta)
WP_028974362.1 (Spirochaeta)
Spirochaetes
WP_268048822.1 (Clostridium)
WP_084052071.1 (Desulfonispora)
Firmicutes
WP_036972486.1 (Pseudoalteromonas)
WP_063703998.1 (Pseudoalteromonas)
WP_062564751.1 (Pseudoalteromonas)
WP_009840649.1 (Pseudoalteromonas)
WP_086744687.1 (Pseudoalteromonas)
WP_126167137.1 (Shewanella)
WP_214508060.1 (Shewanella)
Proteobacteria
WP_087678743.1 (Garciella)
WP_013897975.1 (Methanosalsum)
WP_048141116.1 (Methanosarcina)
WP_011034271.1 (Methanosarcina)
WP_233084063.1 (Methanococcoides)
WP_236622728.1 (Methanococcoides)
Euryarchaeota (n=5) Firmicutes (n=1)
WP_007504495.1 (Caldalkalibacillus)
WP_048601566.1 (Rubeoparvulum)
WP_115220211.1 (Legionella)
WP_028389403.1 (Legionella)
WP_188538908.1 (Paenibacillus)
WP_148451954.1 (Paenibacillus)
WP_091229586.1 (Fontibacillus)
WP_059052600.1 (Paenibacillus)
WP_036622036.1 (Paenibacillus)
WP_128634073.1 (Paenibacillus)
Firmicutes (n=8) Proteobacteria (n=2)
0.5

(G) *frhB* gene

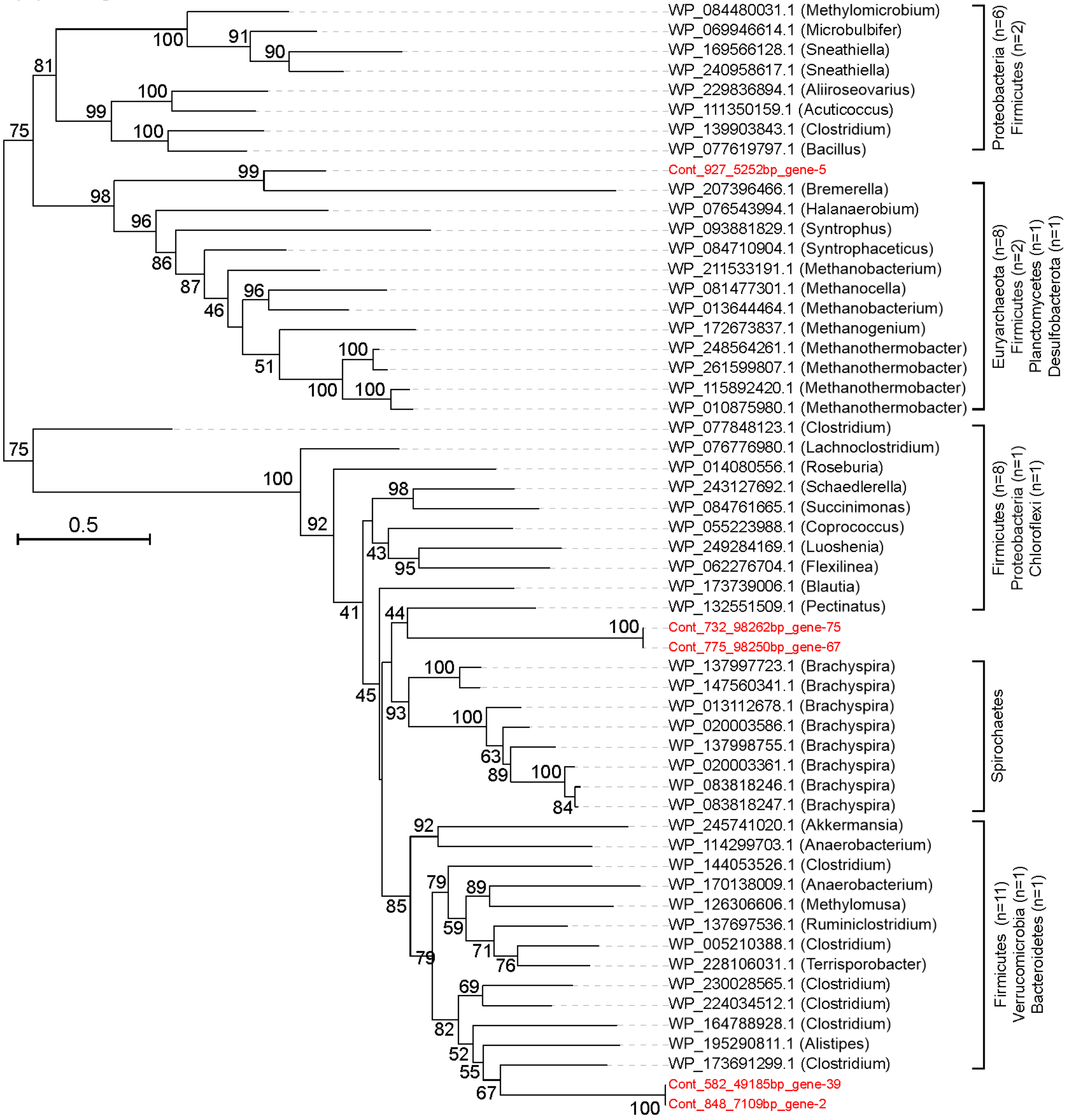

Figure S5. Phylogenetic trees of the seven AMGs that exclusively participate in MMP. The seven AMGs comprised: *mtrA* (A), *pmoC* (B), *fwdF* (C), *fae* (D), *cofE* (E), *cofF* (F), and *frhB* (G). The trees were inferred using maximum likelihood method with protein sequences that showed top hits to each of the studied AMGs (see Methods). Parametric bootstrap values (expressed as percentages of 1000 replications) ≥40 are shown at the branch points. The scale bars indicate a distance as shown in each tree (i.e., 0.1, 0.2, or 0.5 substitutions per position in the alignments). The virus-encoded AMGs identified from this study (all A–G panels) and a previous report (only in B[2]) are indicated in red and light blue, respectively. The microbial sequences obtained from NCBI RefSeq database are indicated black. MMP, methane metabolism pathway.

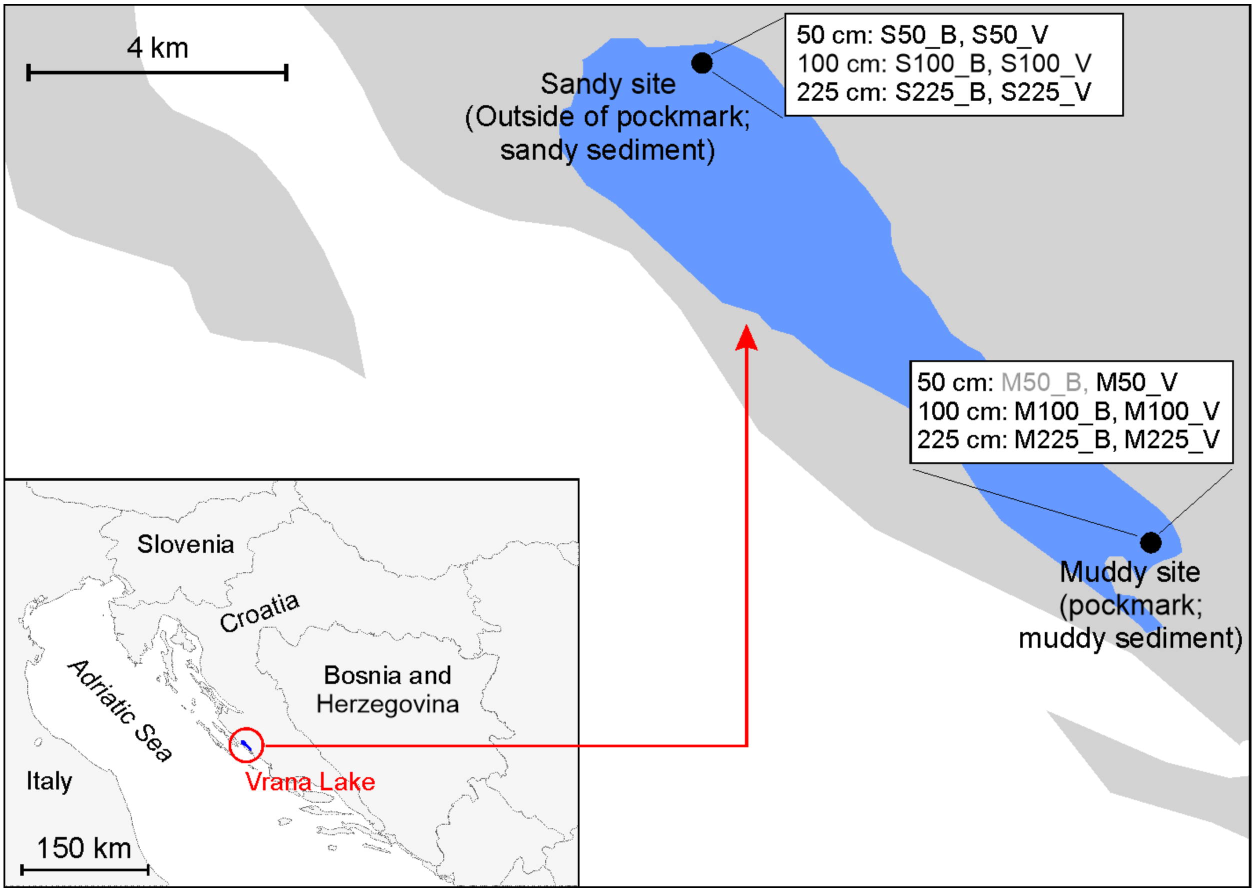

Figure S6. Sampling locations at the Vrana Lake, Croatia. Sediment samples were collected from 50, 100, and 225 cm deep below the lake sediment surface from two sites of the lake: a *muddy site* with muddy sediments in a pockmark depression, indicative of gaseous emissions and a *sandy site* with sandy sediment and no visible pockmark depressions. Each of the six samples was used for constructing a bulk metagenome and a viral metagenome (virome); thus we generated a total of 12 metagenomic libraries: six bulk metagenomes and six viromes, with their sample names provided after each of the sampling depths. The sample names are coded as follows for the examples of S50_B (S, *sandy site*; 50, 50 cm deep; and B, bulk metagenome) and M100_V (M, *muddy site*; 100, 100 cm deep; and V, virome).The sample M50_B failed in sequencing and is indicated in grey. The maps were plotted by the package rnaturalearth (https://github.com/ropensci/rnaturalearth) in R v3.6.1.

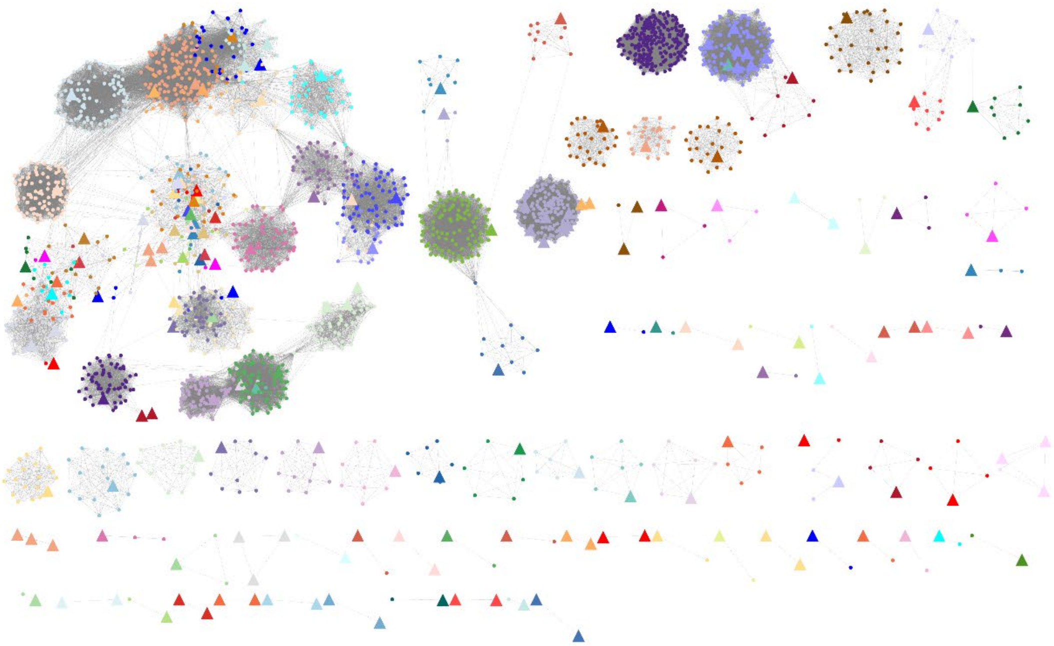

Figure S7. Network clusters of viruses from VLS and databases. Each node represents one viral genome/contig. VLS viruses are indicated by triangles (all with larger size to highlight VLS viruses). Viruses from NCBI RefSeq and IMG/VR databases are included in the network analysis and they were indicated by circles. The edge between nodes represents a significant relationship between two viral genomes/contigs with the shorter lengths accounting for stronger connection strength. Only viruses that formed VCs with VLS viruses are visualized. Viruses within a same VC (total 141 VCs) are indicated by the same color in a cluster. The details of VC clustering and statistical results are provided in Supplementary Data 9. VLS, Vrana Lake Sediment; VC, viral cluster.

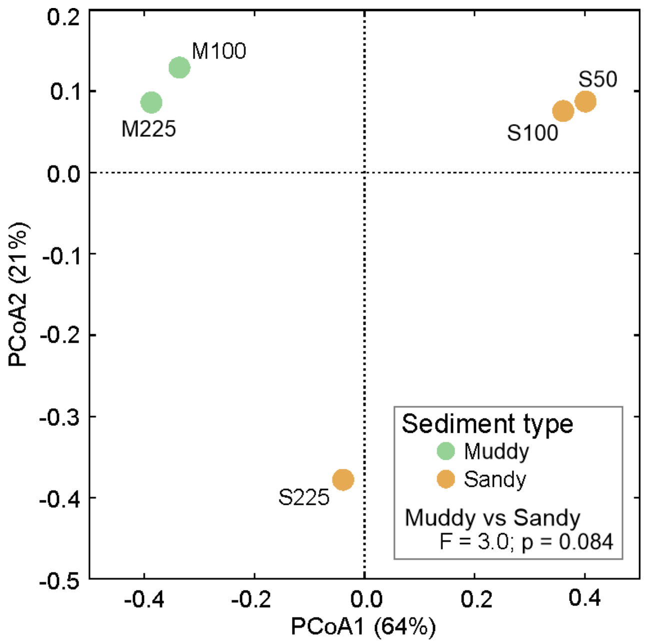

Figure S8. Principal coordinate analysis (PCoA) of microbial community composition. A total of 99 MAGs were recovered from the VLS bulk metagenomes (Supplementary Data 10).The PCoA plot was constructed using Bray-Curtis distance (Source data are provided as a Source Data file), generated based on the relative abundances of MAG populations in the VLS bulk metagenomes (see Methods). Each filled circle represents a sample. Samples from muddy and sandy sediments are colored in light green and orange, respectively. The community difference between sediment types (Muddy vs. Sandy) was assessed by PERMANOVA (Permutational Multivariate Analysis of Variance; permutations = 999) tests. MAG, metagenome-assembled genome; VLS, Vrana Lake sediment.

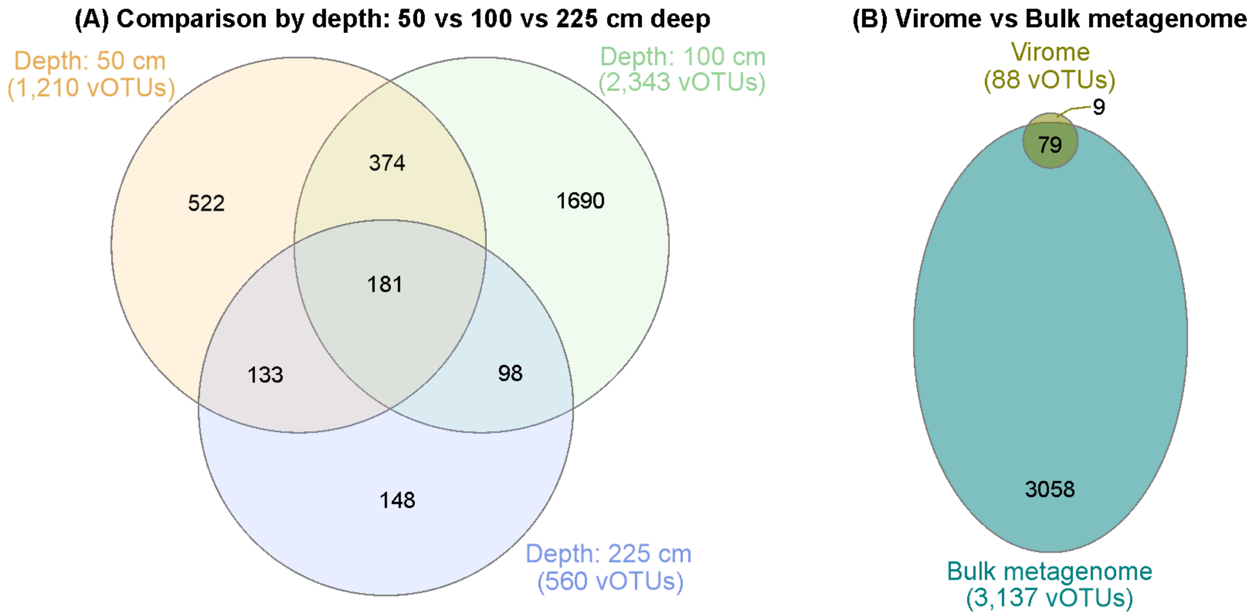

Figure S9. Comparisons of VLS viral communities across sampling depths and between metagenome types. (A) Shared and unique vOTUs among the three sampling depths: 50 vs. 100 vs. 225 cm deep below the lake sediment surface. (B) Shared and unique vOTUs between the two metagenome types: virome vs. bulk metagenome. VLS, Vrana Lake sediment.

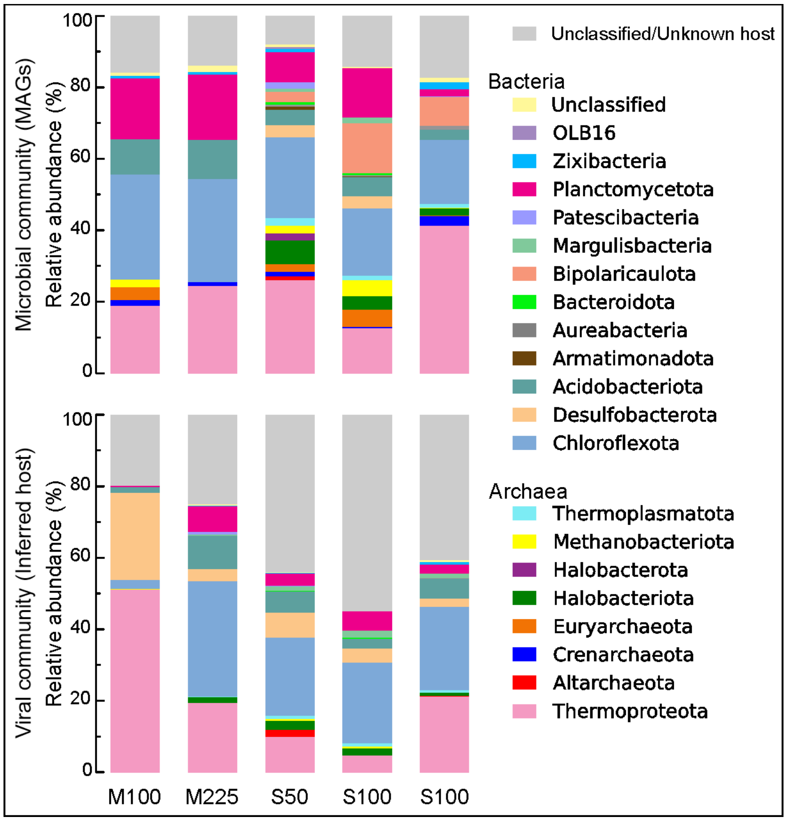

Figure S10. Virus-host linkages and microbial profiles at phylum level. Top bar charts: Microbial community structures constructed according to the relative abundances and taxonomic assignments of MAG populations in VLS bulk metagenomes. Bottom bar charts: Summed relative abundances of VLS vOTUs associated with their predicted hosts (see Methods). VLS, Vrana Lake sediment.

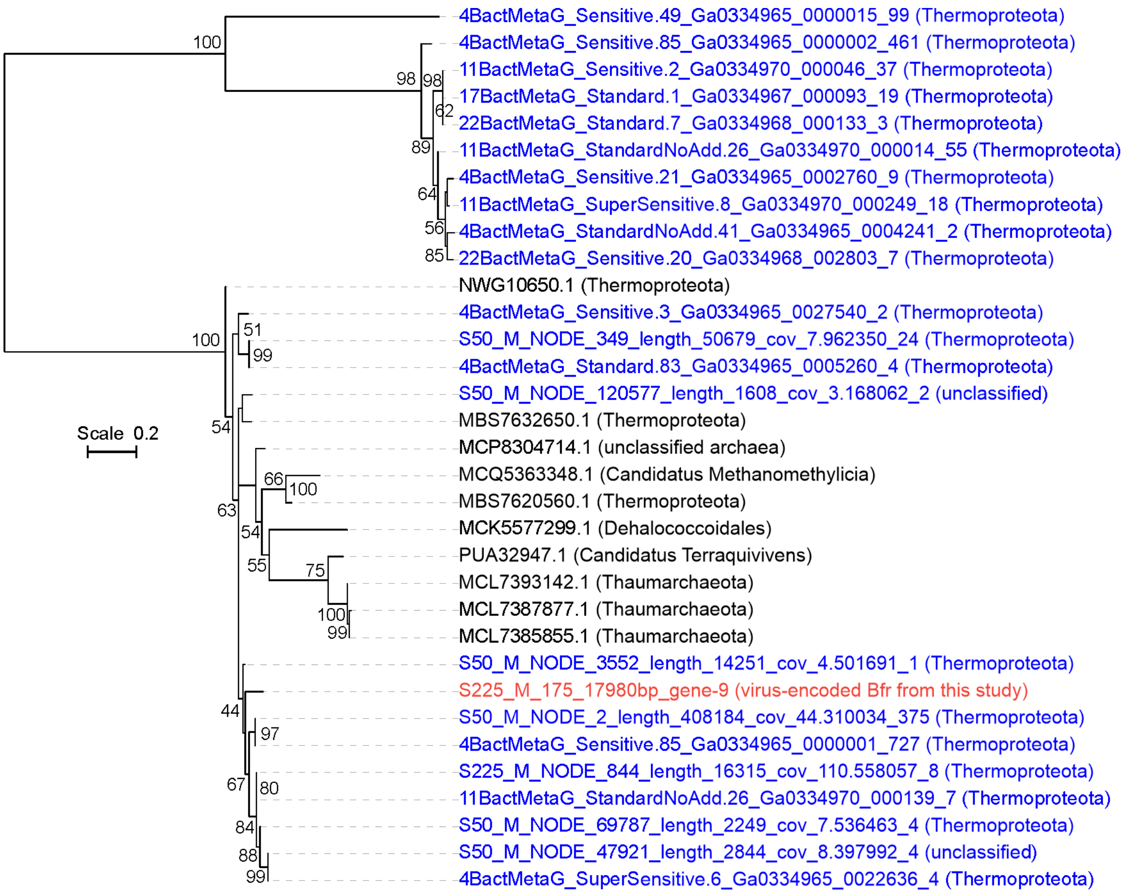

Figure S11. Phylogenetic tree of viral and microbial *bfr* genes. The tree was inferred using maximum likelihood method with protein sequences (see Methods). Bootstrap values (expressed as percentages of 1,000 replications) ≥40 are shown at the branch points. The scale bars indicate a distance of 0.2. The vBfr sequence is indicated in red. The microbial Bfr sequences obtained from the metagenomes of this study and NCBI RefSeq database are indicated in blue and black, respectively. A collapsed version of tree is provided in Fig. 4D.

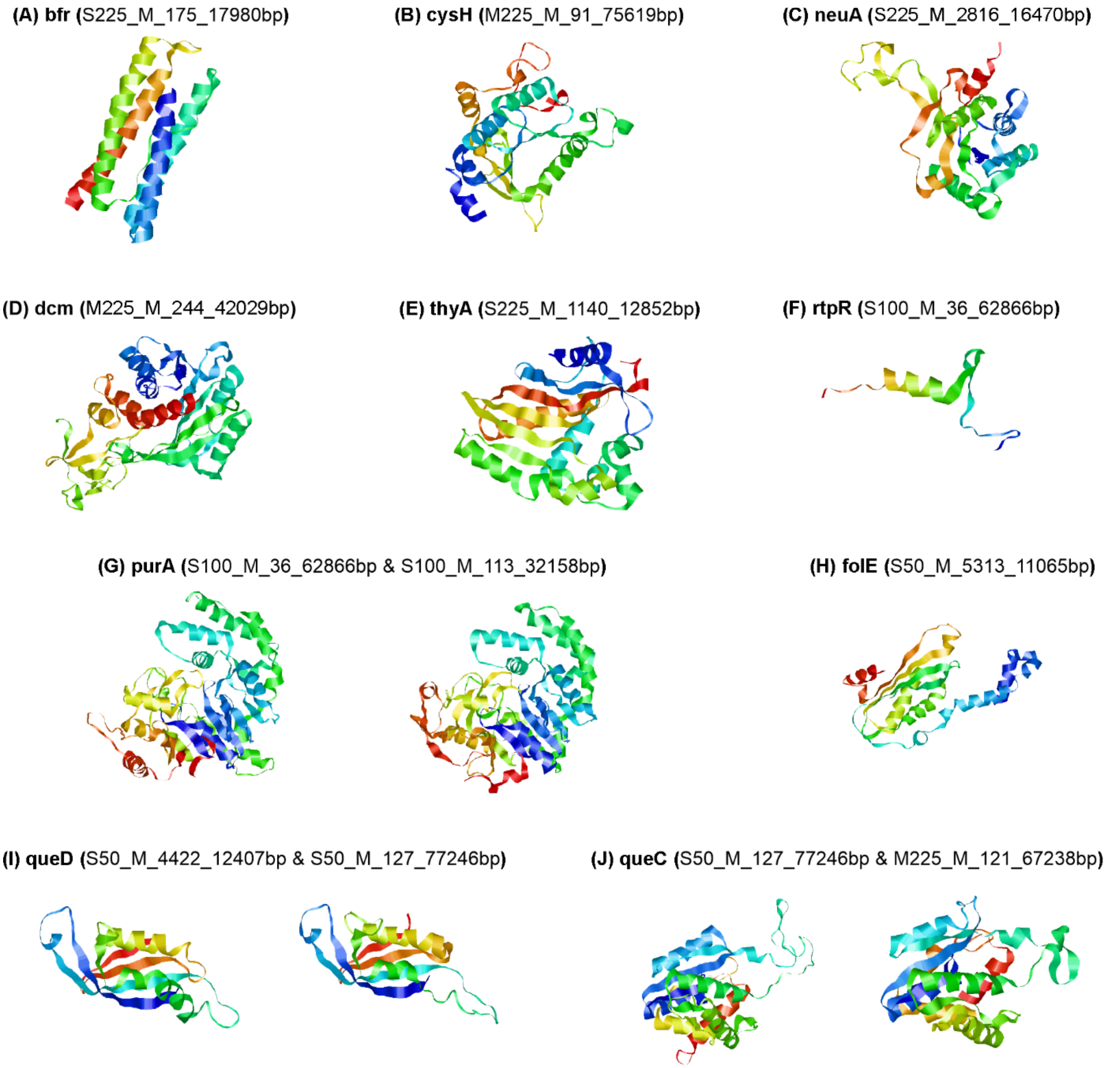

Figure S12. Predicted three-dimensional (3D) structures of the 13 VLS AMG products. These AMGs comprised: *bfr* (A), *cysH* (B), *neuA* (C), *dcm* (D), *thyA* (E), *rtpR* (F), *purA* (G), *folE* (H), *queD* (I), and *queC* (J). All AMG products were linked to their closest template protein with 100% confidence score by Phyre2. More characteristics of these AMGs are summarized in Supplementary Data 15, including the Phyre2 hits. VLS, Vrana Lake sediment.

References:

1 Stewart, R. D. *et al.* Compendium of 4,941 rumen metagenome-assembled genomes for rumen microbiome biology and enzyme discovery. *Nat Biotechnol* **37**, 953-961 (2019).

2 Chen, L. X. *et al.* Large freshwater phages with the potential to augment aerobic methane oxidation. *Nat Microbiol* **5**, 1504-1515 (2020).

3 Latimer, M. T. & Ferry, J. G. Cloning, sequence analysis, and hyperexpression of the genes encoding phosphotransacetylase and acetate kinase from *Methanosarcina thermophila*. *J Bacteriol* **175**, 6822-6829 (1993).

4 González, J. M. & Robb, F. T. Genetic analysis of *Carboxydothermus hydrogenoformans* carbon monoxide dehydrogenase genes *cooF* and *cooS*. *FEMS Microbiol Lett* **191**, 243-247 (2000).

5 Morgavi, D. P., Forano, E., Martin, C. & Newbold, C. J. Microbial ecosystem and methanogenesis in ruminants. *Animal* **4**, 1024-1036 (2010).

6 Schöne, C. & Rother, M. Methanogenesis from Carbon Monoxide. in *Biogenesis of Hydrocarbons* (eds Alfons J. M. Stams & Diana Sousa) 1-29 (Springer International Publishing, 2018).

7 Harrison, E. & Brockhurst, M. A. Ecological and evolutionary benefits of temperate phage: What does or doesn't kill you makes you stronger. *Bioessays* **39**, 1700112 (2017).

8 Knowles, B. *et al.* Lytic to temperate switching of viral communities. *Nature* **531**, 466-470 (2016).

9 Williamson, K. E., Radosevich, M., Smith, D. W. & Wommack, K. E. Incidence of lysogeny within temperate and extreme soil environments. *Environ Microbiol* **9**, 2563-2574 (2007).

10 Trubl, G. *et al.* Soil viruses are underexplored players in ecosystem carbon processing. *mSystems* **3**, e00076-00018 (2018).

11 Emerson, J. B. *et al.* Host-linked soil viral ecology along a permafrost thaw gradient. *Nat Microbiol* **3**, 870-880 (2018).

12 Andrews, S. C., Robinson, A. K. & Rodriguez-Quinones, F. Bacterial iron homeostasis. *FEMS Microbiol Rev* **27**, 215-237 (2003).

13 Andreini, C., Bertini, I., Cavallaro, G., Holliday, G. L. & Thornton, J. M. Metal ions in biological catalysis: from enzyme databases to general principles. *J Biol Inorg Chem* **13**, 1205-1218 (2008).

14 Yang, X., Le Brun, N. E., Thomson, A. J., Moore, G. R. & Chasteen, N. D. The iron oxidation and hydrolysis chemistry of *Escherichia coli* bacterioferritin. *Biochemistry* **39**, 4915-4923 (2000).

15 Yorshansky, O. *et al.* Iron oxides impact sulfate-driven anaerobic oxidation of methane in diffusion-dominated marine sediments. *Front Mar Sci* **9**, 903918 (2022).

16 Cao, X., Wang, Y. & Liu, T. Effects of iron powder addition and thermal hydrolysis on methane production and the archaeal community during the anaerobic digestion of sludge. *Int J Environ Res Public Health* **19**, 4470 (2022).

17 Egger, M. *et al.* Iron-mediated anaerobic oxidation of methane in brackish coastal sediments. *Environ Sci Technol* **49**, 277-283 (2015).
