# Supplementary material for: Viral potential to modulate microbial methane metabolism varies by habitat
Source: Nat Commun. 2024 Feb 29;15:1857. doi: 10.1038/s41467-024-46109-x (PMC10904782; doi:10.1038/s41467-024-46109-x)
Supplement: Supplementary file 3 — Reporting Summary [file 41467_2024_46109_MOESM3_ESM.pdf]

nature portfolio

Corresponding author(s): Matthew B. Sullivan

Last updated by author(s): Jan 15, 2023

Reporting Summary

Nature Portfolio wishes to improve the reproducibility of the work that we publish. This form provides structure for consistency and transparency in reporting. For further information on Nature Portfolio policies, see our Editorial Policies and the Editorial Policy Checklist.

Statistics

For all statistical analyses, confirm that the following items are present in the figure legend, table legend, main text, or Methods section.

n/a	Confirmed	
☐	☒	The exact sample size (*n*) for each experimental group/condition, given as a discrete number and unit of measurement
☐	☒	A statement on whether measurements were taken from distinct samples or whether the same sample was measured repeatedly
☐	☒	The statistical test(s) used AND whether they are one- or two-sided<br>*Only common tests should be described solely by name; describe more complex techniques in the Methods section.*
☐	☒	A description of all covariates tested
☒	☐	A description of any assumptions or corrections, such as tests of normality and adjustment for multiple comparisons
☐	☒	A full description of the statistical parameters including central tendency (e.g. means) or other basic estimates (e.g. regression coefficient) AND variation (e.g. standard deviation) or associated estimates of uncertainty (e.g. confidence intervals)
☐	☒	For null hypothesis testing, the test statistic (e.g. *F*, *t*, *r*) with confidence intervals, effect sizes, degrees of freedom and *P* value noted<br>*Give P values as exact values whenever suitable.*
☒	☐	For Bayesian analysis, information on the choice of priors and Markov chain Monte Carlo settings
☒	☐	For hierarchical and complex designs, identification of the appropriate level for tests and full reporting of outcomes
☒	☐	Estimates of effect sizes (e.g. Cohen's *d*, Pearson's *r*), indicating how they were calculated

Our web collection on statistics for biologists contains articles on many of the points above.

Software and code

Policy information about availability of computer code

Data collection	No software was used for data collection.
Data analysis	VirSorter v1.1.0, DeepVirFinder v1.0, and MARVEL v0.2, BLAST 2.10.0+, nucmer (MUMmer3.23), Bowtie2 2.3.4.3, Read2RefMapper (on CyVerse), vConTACT2 0.9.20, R 3.6.1, SPAdes 3.15.0, MetaBat 2.12.1, GTDB-Tk v1.3.0, CheckM 1.1.10, CheckV 0.3.0, dRep 1.0.0, CoverM 0.3.2, VirMatcher 0.3.3, iPHoP 1.3.2, VIBRANT 1.2.1, DRAM 1.3, NCBI CD-Search v3.2.0, Phyre2 2.0, Easyfig v2.2.5, DIAMOND 2.0.15, MAFFT 7.017, IQ-TREE 1.6.11, iTOL 5, RDP5 5.23, MetaPop v1.0, PAML 4.9, and scripts deposited to GitHub (https://github.com/zhiping393/MM).

For manuscripts utilizing custom algorithms or software that are central to the research but not yet described in published literature, software must be made available to editors and reviewers. We strongly encourage code deposition in a community repository (e.g. GitHub). See the Nature Portfolio guidelines for submitting code & software for further information.

Data

Policy information about availability of data

All manuscripts must include a data availability statement. This statement should provide the following information, where applicable:

- Accession codes, unique identifiers, or web links for publicly available datasets
- A description of any restrictions on data availability
- For clinical datasets or third party data, please ensure that the statement adheres to our policy

All metagenomic data of Vrana Lake sediment samples are available to public via the IMG system with GOLD Project accession codes Gp0356281 (https://

gold.jgi.doe.gov/project?id=Gp0356281), Gp0356283 (https://gold.jgi.doe.gov/project?id=Gp0356283), Gp0356284 (https://gold.jgi.doe.gov/project?id=Gp0356284), Gp0356285 (https://gold.jgi.doe.gov/project?id=Gp0356285), Gp0356286 (https://gold.jgi.doe.gov/project?id=Gp0356286), Gp0356287 (https://gold.jgi.doe.gov/project?id=Gp0356287), Gp0356288 (https://gold.jgi.doe.gov/project?id=Gp0356288), Gp0356289 (https://gold.jgi.doe.gov/project?id=Gp0356289), Gp0356290 (https://gold.jgi.doe.gov/project?id=Gp0356290), Gp0356291 (https://gold.jgi.doe.gov/project?id=Gp0356291), and Gp0356292 (https://gold.jgi.doe.gov/project?id=Gp0356292), as well as via the NCBI Sequence Read Archive (SRA) database with the BioSample accession codes SAMN12796108 (https://www.ncbi.nlm.nih.gov/biosample/?term=SAMN12796108), SAMN14514859 (https://www.ncbi.nlm.nih.gov/biosample/?term=SAMN14514859), SAMN14515366 (https://www.ncbi.nlm.nih.gov/biosample/?term=SAMN14515366), SAMN14515583 (https://www.ncbi.nlm.nih.gov/biosample/?term=SAMN14515583), SAMN14515785 (SAMN14515785), SAMN15738573 (https://www.ncbi.nlm.nih.gov/biosample/?term=SAMN15738573), SAMN18258200 (https://www.ncbi.nlm.nih.gov/biosample/?term=SAMN18258200), SAMN18258201 (https://www.ncbi.nlm.nih.gov/biosample/?term=SAMN18258201), SAMN18259037 (https://www.ncbi.nlm.nih.gov/biosample/?term=SAMN18259037), SAMN18259401 (https://www.ncbi.nlm.nih.gov/biosample/?term=SAMN18259401), and SAMN18261530 (https://www.ncbi.nlm.nih.gov/biosample/?term=SAMN18261530). All the above accession codes are also provided in Table S8 of the Supplementary Data. All the analyzed VLS viral contigs and MAGs, as well as the 911 public data-derived viral contigs containing MM AMGs are available at Figshare (DOI: https://doi.org/10.6084/m9.figshare.23614812). The accession information of publicly available metagenomes used in this study are provided in Table S1 of the Supplementary Data. The following public databases were used for data analyses in this study: KEGG (https://www.genome.jp/kegg/), PFAM (http://pfam.xfam.org/), IMG/VR (https://genome.jgi.doe.gov/portal/IMG_VR/IMG_VR.home.html), NCBI RefSeq (https://ftp.ncbi.nlm.nih.gov/refseq/), and Conserved Domain (https://www.ncbi.nlm.nih.gov/Structure/cdd/wrpsb.cgi) databases.

Research involving human participants, their data, or biological material

Policy information about studies with human participants or human data. See also policy information about sex, gender (identity/presentation), and sexual orientation and race, ethnicity and racism.

Reporting on sex and gender	Not Applicable.
Reporting on race, ethnicity, or other socially relevant groupings	Not Applicable.
Population characteristics	Not Applicable.
Recruitment	Not Applicable.
Ethics oversight	Not Applicable.

Note that full information on the approval of the study protocol must also be provided in the manuscript.

Field-specific reporting

Please select the one below that is the best fit for your research. If you are not sure, read the appropriate sections before making your selection.

☒ Life sciences ☐ Behavioural & social sciences ☐ Ecological, evolutionary & environmental sciences

For a reference copy of the document with all sections, see nature.com/documents/nr-reporting-summary-flat.pdf

Life sciences study design

All studies must disclose on these points even when the disclosure is negative.

Sample size	We analyzed 11 new and 982 publicly available metagenomes. For the 11 new metagenomes, we measured 5 and 6 from pockmark and non-pockmark sites, respectively, of the Vrana Lake, to compare virus communities between the two sites. For the 982 publicly available metagenomes, the sample size was not predetermined; we selected these metagenomes for investigating the potential impacts of viruses on methane metabolism because they were from the environments which are known from literature to potentially host methane-cycling microbial communities, and in which we were able to confirm the presence of microbial MM genes.
Data exclusions	No data were excluded.
Replication	Replication was not applicable due to the technical difficulty in sampling Vrana Lake sediment cores by replicates and the cost limitation for metagenomic sequencing of replicates. However, we sampled 3 depths from each sediment core, and this can be considered as replicates of each sampling site (i.e., pockmark versus non-pockmark sites) for comparing the viral communities between sampling sites.
Randomization	In the metagenomic sequencing and data analyses for identifying viruses and AMGs, the analyst randomly analyzed the lake sediment samples before being informed about the meta data of each sample. However, this is an observational investigation, thus randomization does not impact the main findings of this study.
Blinding	The analyst was blinded with the meta data when analyzing the lake sediment metagenomes for identifying viruses and AMGs (as mentioned above). This is an observational investigation, blinding treatment dose not impact the main findings.

Reporting for specific materials, systems and methods

We require information from authors about some types of materials, experimental systems and methods used in many studies. Here, indicate whether each material, system or method listed is relevant to your study. If you are not sure if a list item applies to your research, read the appropriate section before selecting a response.

Materials & experimental systems

n/a	Involved in the study
☒	☐ Antibodies
☒	☐ Eukaryotic cell lines
☒	☐ Palaeontology and archaeology
☒	☐ Animals and other organisms
☒	☐ Clinical data
☒	☐ Dual use research of concern
☒	☐ Plants

Methods

n/a	Involved in the study
☒	☐ ChIP-seq
☒	☐ Flow cytometry
☒	☐ MRI-based neuroimaging
