# Supplementary material for: Viral potential to modulate microbial methane metabolism varies by habitat
Source: Nat Commun. 2024 Feb 29;15:1857. doi: 10.1038/s41467-024-46109-x (PMC10904782; doi:10.1038/s41467-024-46109-x)
Supplement: Supplementary file 4 — Description of Additional Supplementary files [file 41467_2024_46109_MOESM4_ESM.pdf]

Description of Additional Supplementary files

File name: Supplementary Data 1- 16
Description:

Supplementary Data 1
Description: Summary of the published datasets for recovering virus-encoded AMGs that could be involved in the pathway of methane metabolism. These datasets are from 15 environments (Column B). Data type adapted for analyses in this study: the public data we obtained to initiate the analyses, e.g., "Viral contigs" means that we directly downloaded the viral contigs reported in the public storage for AMG identification, "Assembly contigs" means that we directly downloaded the assembled contigs reported in the public storage for viral identification and AMG identification, while "Metagenome" means that we downloaded the raw metagenomic reads for a series of further analyses including reads quality control, assembly, viral identification, and AMG identification.

Supplementary Data 2
Description: Microbial MM genes in each of the 15 environments. Each habitat contained 138–183 MM genes, as summarized in Supplementary Data 3. MM genes that were also encoded by viruses in the same environment are indicated by "Yes" in Column C. MM, methane metabolism; MMP, methane metabolism pathway.

Supplementary Data 3
Description: Number of distinct microbe- and virus-encoded MM genes in each of the 15 environments. Gene annotations of all microbial MM genes in each environment are listed in Supplementary Data 2. The number of distinct microbe- and virus-encoded MM genes was not significantly correlated by Pearson correlation analysis (R^2 = 0.228, p = 0.062; two-tailed test). MM, methane metabolism.

Supplementary Data 4
Description: Overall summary of putative AMGs involved in the methane metabolism pathway (MMP). The CheckV statistics, viral taxonomy, and putative hosts of viral contigs containing a MM AMG are provided. A total of 24 distinct MM AMGs were identified after rigorous inspection (see Methods), from 911 viral contigs recovered from 6 types of environments: rumen, marine water, marine sediment, lake water, lake sediment, and hot spring. These MM AMGs could potentially participate in 25 reactions of the MMP (Supplementary Fig. S1). The representative contigs (one contig for each AMG) containing the 24 AMGs are summarized in Supplementary Data 5 and their genome maps are illustrated in Supplementary Fig. S2.

Supplementary Data 5
Description: Representative contigs of the 24 AMGs involved in the methane metabolism pathway (MMP). For each of the 24 AMGs, one viral contig was selected for the summary in this table, and for illustrating their genome maps in Supplementary Fig. S2.

Supplementary Data 6
Description: Taxonomic assignments and viral cluster summary for viruses encoding MM AMGs. Viruses from IMG/VR and NCBI RefSeq databases, as well as the 389 ⩾10kb viral contigs encoding MM AMGs, were included for taxonomic analysis. 149 of the 389 viral contigs formed

viral clusters with 1,573 viruses from the databases and all belonged to class Caudoviricetes of phylum Uroviricota, except one virus (i.e., Cont_348_102504bp) that belonged to an unclassified class of the phyla Nucleocytoviricota. Additional 159 viral contigs were clustered into same vOTUs (~species-level viral taxonomic unit) that contained the 149 viral contigs with taxonomy, thus were also assigned to the class Caudoviricetes of phylum Uroviricota. Overall, 308 of 911 MM AMG-carrying viral contigs were assigned to taxonomy. For the viruses from databases, only those forming viral clusters with the MM gene-carrying viruses were summarized in this table. The taxonomic analyses, including the statistical assessments, were conducted by the tool vConTACT v2. MM, methane metabolism.

Supplementary Data 7
Description: Genomic annotations of the putative hosts for viruses containing the MM AMGs. Of the 911 viruses containing MM AMGs, 257 were successfully linked to their hosts (summarized in Supplementary Data 4). This table summarizes the gene annotation of these hosts' genomes. Genes involved in MMP are indicated by "MM gene" in Column E. MM, methane metabolism; MMP, methane metabolism pathway.

Supplementary Data 8
Description: Overall characteristics of the VLS samples and metagenomes. The location of each sample in the lake is illustrated in Supplementary Fig. S6. The sample M50_B was collected but failed in sequencing. The sample names are coded as follows for the examples of S50_B (S, sandy site; 50, 50 cm deep; and B, bulk metagenome) and M100_V (M, muddy site; 100, 100 cm deep; and V, virome). The metagenomic data of each sample was deposited to both NCBI SRA and IMG repositories, with their accession codes provided in Columns N and O, respectively. NA, not appliable or failed test. VLS, Vrana Lake sediment.

Supplementary Data 9
Description: Taxonomic assignments and viral cluster summary of VLS viruses. Viruses from VLS (this study), NCBI RefSeq database, and IMG/VR database were included for taxonomic analysis. Database viruses are listed in this table if they formed viral clusters with VLS viruses. The taxonomic analyses, including the statistical assessments, were conducted by the tool vConTACT v2. VLS, Vrana Lake sediment.

Supplementary Data 10
Description: Taxonomy and genomic quality of the 99 VLS MAGs (metagenome-assembled genomes). NA, not available.

Supplementary Data 11
Description: Relative abundance (%) of vOTUs across all VLS samples. VLS, Vrana Lake sediment.

Supplementary Data 12
Description: Putative microbial hosts of VLS viruses. Of the 3,146 VLS vOTUs, 2,167 were linked to their hosts by the tool VirMatcher using the MAGs obtained from the VLS bulk metagenomes as the host database. Hosts were selected if the "final score" was 1 or larger. VLS, Vrana Lake sediment.

Supplementary Data 13
Description: Gene annotations of 99 VLS MAGs. Genes involved in MMP are indicated by "MM gene" in Column E. MAGs belonging to Thermoproteota are highlighted by grey background

(Rows 3–41,625), and the MAG 11BactMetaG_StandardNoAdd.10, the putative host of the VLS virus containing the AMG *bfr*, is show at Rows 3–1678. MM, methane metabolism; MMP, methane metabolism pathway.

Supplementary Data 14
Description: Putative gene annotations of VLS vOTUs. Blank cells represent "no hit" to the references in the tested databases. VLS, Vrana Lake sediment.

Supplementary Data 15
Description: Overall characteristics of the putative AMGs from VLS viruses. Annotation, conserved domain, protein structure modelling hit, SNP #, and selection pressure value (*pN*/*pS*) of each AMG are summarized. The CheckV statistics and putative hosts (predicted by VirMather) of the viral contigs containing an AMG are provided. Hosts were selected if the "final score" was 0.5 or larger. VLS, Vrana Lake sediment.

Supplementary Data 16
Description: Tests for selection pressure of *bfr* gene using site and free-ratio models. Likelihood ratio test (LRT) was used to evaluate the different comparisons in the hypotheses at 0.05 significance level (one-sided) by Chi-Square tests (pchisq() function in R) using codeml package in PAML.
